# Telehealth Availability for Cancer Care During the COVID-19 Pandemic: Cross-Sectional Study

**DOI:** 10.2196/45518

**Published:** 2023-11-02

**Authors:** Victoria A Marks, Walter R Hsiang, James Nie, Waez Umer, Afash Haleem, Bayan Galal, Irene Pak, Dana Kim, Michelle C Salazar, Haddon Pantel, Elizabeth R Berger, Daniel J Boffa, Jaime A Cavallo, Michael S Leapman

**Affiliations:** 1 Yale School of Medicine New Haven, CT United States; 2 University of California San Francisco School of Medicine San Francisco, CA United States; 3 The College of New Jersey Ewing, NJ United States

**Keywords:** telehealth, colorectal cancer, breast cancer, melanoma, access to care, COVID-19 pandemic, telemedicine, national survey, cross-sectional, cancer, oncology

## Abstract

**Background:**

Telehealth was an important strategy for maintaining continuity of cancer care during the coronavirus pandemic and has continued to play a role in outpatient care; however, it is unknown whether services are equally available across cancer hospitals.

**Objective:**

This study aimed to assess telehealth availability at cancer hospitals for new and established patients with common cancers to contextualize the impact of access barriers to technology on overall access to health care.

**Methods:**

We conducted a national cross-sectional secret shopper study from June to November 2020 to assess telehealth availability at cancer hospitals for new and established patients with colorectal, breast, and skin (melanoma) cancer. We examined facility-level factors to determine predictors of telehealth availability.

**Results:**

Of the 312 investigated facilities, 97.1% (n=303) provided telehealth services for at least 1 cancer site. Telehealth was less available to new compared to established patients (n=226, 72% vs n=301, 97.1%). The surveyed cancer hospitals more commonly offered telehealth visits for breast cancer care (n=266, 85%) and provided lower access to telehealth for skin (melanoma) cancer care (n=231, 74%). Most hospitals (n=163, 52%) offered telehealth for all 3 cancer types. Telehealth availability was weakly correlated across cancer types within a given facility for new (r=0.16, 95% CI 0.09-0.23) and established (r=0.14, 95% CI 0.08-0.21) patients. Telehealth was more commonly available for new patients at National Cancer Institute–designated facilities, medical school–affiliated facilities, and major teaching sites, with high total admissions and below-average timeliness of care. Telehealth availability for established patients was highest at Academic Comprehensive Cancer Programs, nongovernment and nonprofit facilities, medical school–affiliated facilities, Accountable Care Organizations, and facilities with a high number of total admissions.

**Conclusions:**

Despite an increase in telehealth services for patients with cancer during the COVID-19 pandemic, we identified differences in access across cancer hospitals, which may relate to measures of clinical volume, affiliation, and infrastructure.

## Introduction

The COVID-19 pandemic disrupted the delivery of cancer care around the world [1,2]. For at least some period of time, most patients were unable to receive in-person care due to pandemic-related hospital restrictions and exposure risks. These delays are expected to have significant downstream effects—modeling studies have estimated a 15%-16% increase in deaths due to colorectal cancer and an 8%-10% increase in deaths due to breast cancer in the postpandemic period up to 5 years after diagnosis [3].

To maintain continuity of care during the pandemic, alternative mediums of health care delivery were used, primarily telehealth [4]. Although telehealth has long been available, most physicians did not offer telehealth services prior to the COVID-19 pandemic [5,6]. Due to the urgent need for remote provision of health care services triggered by the COVID-19 pandemic, government and health care providers temporarily removed reimbursement and access barriers and enhanced facility infrastructure [7,8]. As a result, telehealth use dramatically increased, with a 50- to 175-fold increase in the number of patients seen via telehealth compared to prepandemic practice [9,10]. In this way, the adoption of a technological platform, telehealth, served as a solution for the problem of access to health care generated by the COVID-19 pandemic crisis.

Despite the increased use of telehealth, the extent of access to telehealth for cancer care at a facility level during the COVID-19 pandemic is unknown. Although there has been a rapid proliferation of studies addressing telehealth during the pandemic, most existing studies addressing cancer care have not analyzed facility-level characteristics and telehealth uptake [10-18]. Therefore, we aimed to assess telehealth availability for cancer care in the United States during the COVID-19 pandemic at facilities recognized for cancer care excellence with the goal of understanding factors associated with initial uptake. We chose to investigate cancers with early treatment interventions—colorectal, breast, and skin (melanoma) cancer—as delays in health care services due to COVID-19 have been projected to have enduring downstream consequences. We hypothesized that despite increases in the use of telehealth during the COVID-19 pandemic, disparities in access to telehealth for cancer care persisted.

## Methods

### Study Sample and Data

The primary objective of this study was to characterize telehealth availability for cancer care for patients with colorectal, breast, or skin (melanoma) cancer. In addition, we investigated characteristics of facilities that provide high telehealth access for cancer care, defined as the provision of telehealth appointments for all 3 investigated cancer types. We examined telehealth availability by cancer site and separately evaluated access for new and established patients.

We conducted a national cross-sectional secret shopper study from June 3 to November 9, 2020. Secret shopper studies can effectively assess access to care from the patient's perspective by using simulated patient calls to physician offices to attempt to schedule appointments for surgical consults [19-23]. Trained investigators contacted specialty departments at identified facilities, posing as an individual seeking care for a family member (simulated patient) with a new cancer diagnosis. Institutions were not notified of the simulated patient call prior to the investigation, and no real patient information was used for the purpose of this study. Investigators recorded department referral location, telehealth availability for new patients (ie, initial appointment availability), and telehealth availability for established patients (ie, follow-up visit availability).

### Variable Measures

We identified cancer care facilities using the American College of Surgeon’s Commission on Cancer Hospital Locator [24]. We excluded facilities with unique membership policies, as such policies are likely to skew facility-level characteristics and subsequent analysis. These facilities included Veterans Affairs and Kaiser Foundation hospitals; specialty programs, such as pediatric cancer, hospital associate cancer, freestanding cancer, oncology medical home, and rectal cancer–only programs; and facilities located in Puerto Rico. We then used a random number generator to create a representative sample of approximately one-third of eligible facilities.

We characterized facilities included in the sample using the 2016 American Hospital Association Annual Survey database and the publicly available Centers for Medicare and Medicaid Services (CMS) General Information database [25,26]. We investigated facility characteristics known to influence health care access and outcomes, including organization infrastructure, financials, and services provided. Example characteristics include types of cancer programs, ownership, medical school affiliation, major teaching hospital, Accountable Care Organization, and total facility admissions. Types of cancer programs include Community (facilities seeing <500 and >100 newly diagnosed cancer cases annually), Comprehensive Community (facilities seeing >500 cases annually), Academic Comprehensive (facilities seeing >500 cases annually, with postgraduate medical education provided), Integrated Network (multifacility systems with integrated, comprehensive cancer services), and National Cancer Institute (NCI)–designated cancer programs (facilities with NCI Cancer Center Support Grants) [24]. The CMS database provides information on facility performance, including overall rating, the effectiveness of care, and timeliness of care, defined as how often and quickly hospitals provide care shown to yield the best outcomes for patients with certain conditions (eg, cancer care, colonoscopy follow-up, preventative care, and sepsis care) [27].

We excluded facilities where at least 1 specialty department of interest was unable to be contacted as well as facilities that were not included in both the American Hospital Association and CMS databases.

### Data Analysis Procedure

The primary study outcome was telehealth appointment availability for new and established patients with a presumptive cancer diagnosis (available vs not available). To evaluate whether the availability of telehealth services for 1 cancer type was associated with others within a given institution, we used a two-way mixed effects model with absolute agreement to determine single measures intraclass correlation coefficients. Additionally, we assessed facility characteristics associated with high access to telehealth for new and established patients. We used chi-square tests to evaluate associations between facility characteristics and telehealth access (*P*<.05 was considered statistically significant). We redefined continuous variables into quintiles and compared the highest quintile against the lowest 4 quintiles. The statistical analyses were performed using JMP 15 (SAS Institute) and IBM SPSS Statistics for Windows (version 28.0.0.0; IBM Corp). Facility locations and their telehealth appointment availability were mapped using ArcGIS software by Esri.

### Ethical Considerations

The Yale School of Medicine Institutional Review Board deemed this study exempt from review (IRB #2000030368). This study was not identified as a human subject research.

## Results

We contacted 312 Commission on Cancer (CoC)–accredited facilities for each of the 3 investigated cancer types, representing 27% of all facilities that met inclusion criteria. Overall, 97.1% (n=303) of facilities provided some form of telehealth for patients with cancer. At the time of the interview, 72.4% (n=226) of surveyed facilities offered new telehealth services for at least 1 cancer type, 39.1% (n=122) for at least 2, and 10.9% (n=34) for all 3 cancer types surveyed. Comparatively, 97.1% (n=303) offered telehealth for established patients for at least 1 cancer type, 85.3% (n=266) for at least 2, and 51.6% (n=161) for all 3 cancer types. Telehealth appointments for new versus established patients were offered at 39.7% (n=124) versus 74.4% (n=232) of facilities for colorectal cancer, 35.6% (n=111) versus 85.3% (n=266) of facilities for breast cancer, and 47.1% (n=147) versus 73.7% (n=230) for skin cancer care. Telehealth was not offered in 24.4% (n=76) of facilities for colorectal, 14.7% (n=46) for breast, and 26.0% (n=81) for skin cancer care (Table 1).

**Table 1:** Telehealth appointment availability for new and established patient visits in the United States during the COVID-19 pandemic (June-November 2020).

Telehealth appointment availability	All cancer types, n (%)	Colorectal cancer, n (%)	Breast cancer, n (%)	Skin cancer, n (%)
New patients	34 (10.9)	124 (39.7)	111 (35.6)	147 (47.1)
Established patients	161 (51.6)	232 (74.4)	266 (85.3)	230 (73.7)
Both new and established patients	32 (10.3)	120 (38.5)	111 (35.6)	146 (46.8)
No appointments available	149 (47.8)	76 (24.4)	46 (14.7)	81 (26.0)
Only new patients (no established patients)	2 (0.6)	4 (1.3)	0 (0)	1 (0.3)
Only established patients (no new patients)	129 (41.3)	112 (35.9)	155 (49.7)	84 (26.9)
Any form of telehealth appointment availability offered	163 (52.2)	236 (75.6)	266 (85.3)	231 (74.0)

**Figure 1:**
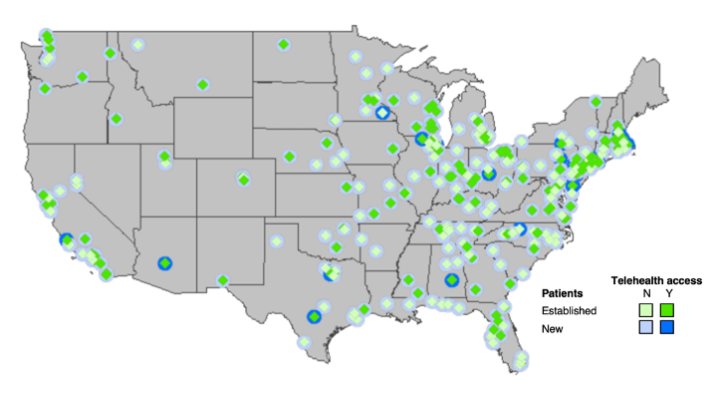
Telehealth availability at cancer care facilities across the United States. State boundary data were extracted from the state (generalized) publicly available data set. N: No access to telehealth. Y: access to telehealth. Green: established patients. Blue: new patients.

Figure 1 shows the geographic distribution of high telehealth access facilities offering telehealth appointments for new and established patients for all investigated cancer types. Of note, even in regions with a lower density of cancer care facilities, few centers offered telehealth services for new patients with cancer.

When examining facilities providing high access to telehealth or access to telehealth appointments for all 3 investigated cancer types, 10.9% (n=34) provided high access for new patients, 51.6% (n=161) provided high access for established patients (Table 1), and 47.8% (n=149) of facilities offered no uniform telehealth availability for all cancer types (Table 1). Only 10.3% (n=32) of facilities offered telehealth appointments for both new and established patients for all 3 cancer types (Table 1). Although 41.3% (n=129) of facilities offered telehealth for established patients for all cancer types, less than 1% (n=2) of facilities offered telehealth for only new patients for all cancer types (Table 1). The correlation of telehealth availability across cancer types within facilities was weak for both new (*r=*0.16, 95% CI 0.09-0.23) and established patients (*r=*0.14, 95% CI 0.08-0.21).

Facility characteristics by telehealth access status are detailed in Table 2.

**Table 2:** Characteristics of facilities with high access to telehealth for new and established patient visits in the United States during the COVID-19 pandemic (June-November 2020). Statistically significant *P* values (*P*<.05) are italicized.

Characteristics		Total facilities, n (%)	Facilities with high access to telehealth for new patients				Facilities with high access to telehealth for established patients			
			Total, n (%)	*P* value		Total, n (%)			*P* value	
**Type of cancer care program**					*<.001*					*.02*
	Community	74 (23.7)	2 (2.7)			30 (40.5)				
	Comprehensive Community	146 (46.8)	11 (7.5)			71 (48.6)				
	Academic Comprehensive	44 (14.1)	11 (25.0)			30 (68.2)				
	Integrated Network	36 (11.5)	5 (13.9)			23 (63.9)				
	NCI^a^ designated	12 (3.8)	5 (41.7)			7 (58.3)				
**Ownership**					.24					*.03*
	For-profit	38 (12.2)	2 (5.3)			14 (36.8)				
	Nongovernment, nonprofit	239 (76.6)	30 (12.6)			133 (55.6)				
	Government	35 (11.2)	2 (5.7)			14 (40.0)				
**Medical school affiliation**					*.003*					*.002*
	No	119 (38.1)	5 (4.2)			48 (40.3)				
	Yes	193 (61.9)	29 (15.0)			113 (58.5)				
**Major teaching hospital**					*<.001*					*<.001*
	No	254 (81.4)	17 (6.7)			119 (46.9)				
	Yes	58 (18.6)	17 (29.3)			42 (72.4)				
**Accountable Care Organization**					.18					*.04*
	No	120 (43.6)	10 (8.3)			54 (45.0)				
	Yes	155 (56.4)	22 (14.2)			90 (58.1)				
**Total facility admissions**					*<.001*					*.002*
	Lowest 4 quintiles	256 (82.3)	20 (7.8)			122 (47.7)				
	Highest quintile	55 (17.7)	14 (25.5)			39 (70.9)				
**Hospital overall rating**					.15					.90
	1 star (lowest)	20 (6.5)	1 (5)			12 (60.0)				
	2 stars	72 (23.2)	12 (16.7)			35 (48.6)				
	3 stars	77 (24.8)	9 (11.7)			38 (49.4)				
	4 stars	93 (30.0)	5 (5.4)			48 (51.6)				
	5 stars (highest)	48 (15.5)	7 (14.6)			26 (54.2)				
**Effectiveness of care**					.24					.07
	Below national average	30 (9.7)	6 (20.0)			21 (70)				
	Same as national average	266 (86.1)	27 (10.2)			133 (50)				
	Above national average	13 (4.2)	1 (7.7)			5 (38.5)				
**Timeliness of care**					*.006*					.10
	Below national average	159 (51.5)	26 (16.4)			91 (57.2)				
	Same as national average	104 (33.7)	7 (6.7)			46 (44.2)				
	Above national average	46 (14.9)	1 (2.2)			22 (47.8)				

^a^NCI: National Cancer Institute.

The sample mostly consisted of nongovernment, nonprofit facilities (239, 76.6%); medical school–affiliated facilities (193, 61.9%); and nonmajor teaching facilities (254, 81.4%). For new patients, NCI-designated facilities offered high access to telehealth (5/12, 41.7%), while Community Cancer Programs had the lowest access to telehealth (2/74, 2.7%; *P*<.001). Medical school–affiliated facilities (29/193, 15.0% vs 5/119, 4.2%; *P*=.003), major teaching facilities (17/58, 29.3% vs 17/254 6.7%; *P*<.001), and facilities in the highest quintile of total admissions (14/55, 25.5% vs 20/256, 7.8%; *P*<.001) were significantly more likely to offer telehealth to new patients compared to facilities not affiliated with medical schools. Facilities with below-average timeliness of care (26/159, 16.4%) were also more likely to offer telehealth to all new patients compared to those with average (7/104, 6.7%) or above average (1/46, 2.2%) timeliness of care (*P*=.006).

Telehealth availability for all cancer types for established patients also significantly differed by cancer program, with Academic Comprehensive Cancer Programs most frequently offering high telehealth access (30/44, 68.2%), followed by Integrated Network (23/36, 63.9%), NCI-designated facilities (7/12, 58.3%), Comprehensive Community Cancer Program (71/146, 48.6%), and Community Cancer Program (30/74, 40.4%; *P*=.02). Nongovernment, nonprofit facilities (133/239, 55.6%) were more likely to offer high telehealth access compared to government-owned (14/35, 40.0%) and for-profit (14/38, 36.8%) facilities (*P*=.03). Medical school–affiliated facilities (113/193, 58.5% vs 48/119, 40.3%; *P*=.002), major teaching hospitals (42/58, 72.4% vs 119/254, 46.9%; *P*<.001), Accountable Care Organizations (90/155, 58.1% vs 54/120, 45.0%; *P*=.04), and facilities in the highest quintile of total admissions (39/55, 70.9% vs 122/256, 47.7%; *P*=.002) were also more likely to offer high access to telehealth services compared to facilities not affiliated with medical schools. There was no significant difference in telehealth access for new or established patients with varying overall hospital ratings or effectiveness of care ratings (Table 2).

## Discussion

### Principal Findings

Our findings from a national, cross-sectional secret shopper study indicate inconsistent access to telehealth services for patients with cancer during the initial period of the COVID-19 pandemic. Although nearly half of facilities offered access to telehealth services for at least colorectal, breast, or skin cancer care, only 11% (n=34) of facilities offered telehealth appointments for all patients across all 3 cancer types. Moreover, the availability of telehealth was only weakly correlated at the facility level, suggesting that access differences may exist between departments within facilities. Telehealth services were less accessible for new compared to established patients. Finally, we found that NCI-designated cancer centers, facilities with medical school affiliations, teaching hospitals, and higher-volume facilities were more likely to offer telehealth.

We found that access to telehealth varied both between and within facilities. Nearly half of the sampled facilities offered no telehealth for new or established patients with colorectal, breast, or skin cancer during the initial peak of the COVID-19 pandemic. This finding suggests that despite meaningful federal, state, and institutional-level policy initiatives to improve access to telehealth services during the COVID-19 pandemic, significant barriers to access persisted. Further, we found that access was weakly correlated across different cancer types within a given facility. High variation within facilities suggests at least some degree of decentralization and may imply room for shared policies within institutions to standardize access. The literature suggests that similar trends exist in the variation of access to in-person visits across departments for patients with cancer, although this may not specifically apply to new versus established patient populations [23].

Before the COVID-19 pandemic, reimbursement, interstate medical licensure, and access to necessary technology platforms were recognized as key barriers to telehealth adoption [28-31]. The COVID-19 pandemic catalyzed a rapid transformation of telehealth use nationally [9,10]. Federal and state legislation worked to ameliorate some of the key access barriers by broadening reimbursement eligibility for qualifying encounters, waiving or limiting cost-sharing, requiring reimbursement parity for telehealth and in-person services, and expanding practitioner telehealth jurisdiction, with private insurance companies largely following suit [7,8,32]. Facilities also quickly scaled up capabilities to support the shift to remote health care delivery. However, the findings from this study reveal that these initiatives did not eradicate at least the initial barriers to telehealth. Persistent issues barring telehealth access for patients during the pandemic may have included reimbursement—as policies often vary by state—and the facility-level startup costs of telehealth implementation, both financially and administratively [7,33]. This is in addition to patient-driven and socioeconomic barriers, such as patient interest, lack of access to appropriate technology platforms, understanding of the use of technology, and access to safe and private spaces to attend a telehealth interview [33]. Of note, although there are several initiatives in place to continue to enable and broaden the scope of telehealth practice, including the Omnibus FY 2022 Spending Bill, which extends Medicare telehealth flexibilities and coverage, many of the policies implemented to expand telehealth accessibility were temporary, with legislation now or soon to be expired [7,34,35]. To ensure sustained access to telehealth services, barriers to reimbursement, licensure, and technological platforms must be more permanently addressed.

Another key finding of this study is that telehealth appointment availability was significantly lower for new compared to established patients, even in areas with lower density of cancer care facilities, where in-person care may be even more difficult. This finding is in line with telehealth reimbursement expansion policies, such as Medicare waiver 1135, which originally did not extend to new patient visits, suggesting that telehealth availability is largely driven by insurance and reimbursement policies [7,32]. The accuracy of data collection, specifically via observation and physical exams, has also been cited as a concern with telehealth use by providers, given the consequential reluctance to establish surgical treatment plans based on the initial remote visit [33].

We also found that the Academic Comprehensive Cancer Program, medical school–affiliated facilities, major teaching hospitals, and facilities with greater admissions had greater access to telehealth for both new and established patients. These findings are consistent with prior studies, which have shown greater telehealth use among teaching hospitals [36,37]. Despite delivering most cancer care in the United States, Community and Comprehensive Community Cancer Programs provide the lowest access to telehealth services [24]. Reduced availability may be related to smaller institution size, smaller infrastructure, and fewer resources to rapidly implement telehealth.

This study, which broadens our understanding of the early uptake of telehealth services during the COVID-19 pandemic, is relevant for several reasons. First, disparities in accessibility of telehealth services may be indicative of persistent barriers to accessing care. Second, the study focuses attention on areas with the greatest interruption in care. Lastly, it indicates gaps in the infrastructure necessary to facilitate flexibility of health care delivery during health emergencies. Our findings underscore that despite improvements in access to the telehealth landscape during the COVID-19 pandemic, barriers to telehealth persist and identify potential sources of disparities in access to cancer care. These findings suggest that CoC centers may benefit from a more centralized approach to the provision of telehealth services. Improving access to telehealth, particularly during times of increased access barriers to health care (eg, social distancing mandate during a global pandemic), is important, as it has been shown to improve rates of early diagnosis, patient compliance, and treatment retention in addition to patient satisfaction [38-42]. As such, the risk factors highlighted in this study may be considered when constructing telehealth policies and implementation strategies. Future studies should evaluate trends in telehealth use throughout and beyond the COVID-19 pandemic. As with any study, these findings should be considered in the context of potential limitations. Because there was no reference study prior to the COVID-19 pandemic, it is difficult to assess the extent to which telehealth availability was directly affected by the pandemic. However, analysis of both prepandemic and peripandemic telehealth use supports a significant increase in telehealth use during the pandemic [9]. In a similar manner, the telehealth landscape rapidly evolved over the course of the pandemic. As such, telehealth policies for given institutions may have changed over the course of data collection and may not be reflected in the data. Additionally, it is important to note that these data reflect surgical care specifically and do not reflect telehealth availability for cancer care provided by other specialties. The data also do not reflect patient or staff factors affecting telehealth access nor does it reflect the number of treating surgeons at each site, although admissions volume may serve as a surrogate. Finally, our sample was drawn from CoC-accredited facilities, and therefore, does not necessarily represent telehealth access at all sites, with most facilities geographically concentrated in the eastern United States. However, as most of the cancer care is delivered at CoC facilities, we believe that this sample is likely to reflect early patterns of telehealth access for cancer patients during the pandemic.

### Conclusions

In conclusion, in this national cross-sectional study, we assessed telehealth availability across cancer types during the COVID-19 pandemic. We found that 97.1% (n=303) of facilities provided some form of telehealth availability, although only 52.2% (n=163) offered telehealth for colorectal, breast, and skin (melanoma) cancer. We identified differences in the characteristics of facilities that offered access to telehealth for high-access centers, or facilities offering telehealth appointments for the 3 cancer types surveyed, including medical school–affiliated and higher-volume centers. We also uncovered substantial variation in early telehealth availability within cancer hospitals, suggesting that access to telehealth may not be centralized within facilities. Taken together, these findings highlight disparities in access to cancer care services during a national crisis when access to health care services was limited. They also highlight potential pitfalls that may be better addressed in future crises requiring the rapid upscale of technological health care platforms.

## Abbreviations

CMS: Centers for Medicare and Medicaid Services
CoC: Commission of Cancer
NCI: National Cancer Institute

## Data Availability

The data sets generated and analyzed during this study are not publicly available due to the use of proprietary American Hospital Association data and identification of specific health care organizations within the investigator-collected data set but are available from the corresponding author on reasonable request.
